# Supplementary material for: Modeling glioblastoma heterogeneity as a dynamic network of cell states
Source: Mol Syst Biol. 2021 Sep 16;17(9):e10105. doi: 10.15252/msb.202010105 (PMC8444284; doi:10.15252/msb.202010105)
Supplement: Supplementary file 5 — Source Data for Figure 3 [file MSB-17-e10105-s001.zip › Figure3A_sourcedata/GSEA_3065/nosigdb_state3.GseaPreranked.1622718426028/RICHARDS_NOWAKOWSKI_SCIENCE_2017_NIN4_UPREG.html]

Details for gene set RICHARDS\_NOWAKOWSKI\_SCIENCE\_2017\_NIN4\_UPREG[GSEA]

|  || Dataset | state3 |
| Phenotype | NoPhenotypeAvailable |
| Upregulated in class | na\_neg |
| GeneSet | RICHARDS\_NOWAKOWSKI\_SCIENCE\_2017\_NIN4\_UPREG |
| Enrichment Score (ES) | -0.40261528 |
| Normalized Enrichment Score (NES) | -1.5029383 |
| Nominal p-value | 0.0059970017 |
| FDR q-value | 0.05384099 |
| FWER p-Value | 0.8 |
Table: GSEA Results Summary

  

Fig 1: Enrichment plot: RICHARDS\_NOWAKOWSKI\_SCIENCE\_2017\_NIN4\_UPREG      
 Profile of the Running ES Score & Positions of GeneSet Members on the Rank Ordered List

  

| PROBE | GENE SYMBOL | GENE\_TITLE | RANK IN GENE LIST | RANK METRIC SCORE | RUNNING ES | CORE ENRICHMENT || 1 | APLP2 |  |  | 20 | 0.506 | 0.0217 | No |
| 2 | CANX |  |  | 27 | 0.479 | 0.0438 | No |
| 3 | WLS |  |  | 58 | 0.407 | 0.0597 | No |
| 4 | SOX6 |  |  | 88 | 0.358 | 0.0733 | No |
| 5 | SOX11 |  |  | 117 | 0.325 | 0.0855 | No |
| 6 | SNTG1 |  |  | 140 | 0.314 | 0.0979 | No |
| 7 | OCIAD1 |  |  | 151 | 0.310 | 0.1114 | No |
| 8 | CHD7 |  |  | 172 | 0.298 | 0.1233 | No |
| 9 | PRRC2C |  |  | 224 | 0.272 | 0.1303 | No |
| 10 | MEG3 |  |  | 258 | 0.260 | 0.1389 | No |
| 11 | HLTF |  |  | 306 | 0.241 | 0.1449 | No |
| 12 | PCM1 |  |  | 325 | 0.237 | 0.1540 | No |
| 13 | ELOVL5 |  |  | 450 | 0.209 | 0.1496 | No |
| 14 | NDFIP1 |  |  | 464 | 0.206 | 0.1579 | No |
| 15 | TRAM1 |  |  | 519 | 0.196 | 0.1610 | No |
| 16 | HIPK2 |  |  | 545 | 0.193 | 0.1672 | No |
| 17 | AKAP12 |  |  | 640 | 0.180 | 0.1649 | No |
| 18 | SMIM7 |  |  | 765 | 0.161 | 0.1582 | No |
| 19 | HNRNPH1 |  |  | 793 | 0.158 | 0.1626 | No |
| 20 | HSPA4 |  |  | 824 | 0.155 | 0.1665 | No |
| 21 | ANKRD11 |  |  | 996 | 0.138 | 0.1532 | No |
| 22 | TMEM41B |  |  | 1029 | 0.136 | 0.1560 | No |
| 23 | TMEM123 |  |  | 1047 | 0.134 | 0.1604 | No |
| 24 | PCDH17 |  |  | 1059 | 0.134 | 0.1655 | No |
| 25 | TMEM245 |  |  | 1072 | 0.133 | 0.1704 | No |
| 26 | PGM3 |  |  | 1133 | 0.127 | 0.1695 | No |
| 27 | TMTC3 |  |  | 1214 | 0.120 | 0.1659 | No |
| 28 | DDX46 |  |  | 1276 | 0.116 | 0.1644 | No |
| 29 | SACS |  |  | 1360 | 0.111 | 0.1600 | No |
| 30 | VPS35 |  |  | 1421 | 0.107 | 0.1581 | No |
| 31 | EXOC1 |  |  | 1589 | 0.098 | 0.1434 | No |
| 32 | AHCTF1 |  |  | 1693 | 0.092 | 0.1359 | No |
| 33 | CDK5RAP3 |  |  | 1835 | 0.085 | 0.1235 | No |
| 34 | NCOR1 |  |  | 1844 | 0.084 | 0.1266 | No |
| 35 | EPHA4 |  |  | 1881 | 0.082 | 0.1263 | No |
| 36 | CEP78 |  |  | 1933 | 0.079 | 0.1241 | No |
| 37 | GFPT1 |  |  | 1937 | 0.079 | 0.1275 | No |
| 38 | CCNH |  |  | 1991 | 0.076 | 0.1250 | No |
| 39 | ESCO1 |  |  | 2014 | 0.075 | 0.1261 | No |
| 40 | EXOSC6 |  |  | 2277 | 0.065 | 0.0988 | No |
| 41 | MARK3 |  |  | 2328 | 0.063 | 0.0960 | No |
| 42 | GAD1 |  |  | 2334 | 0.063 | 0.0984 | No |
| 43 | KALRN |  |  | 2432 | 0.059 | 0.0900 | No |
| 44 | SEMA6D |  |  | 2534 | 0.056 | 0.0809 | No |
| 45 | MCMBP |  |  | 2581 | 0.054 | 0.0782 | No |
| 46 | DDHD1 |  |  | 2634 | 0.052 | 0.0746 | No |
| 47 | MRPL39 |  |  | 2718 | 0.050 | 0.0673 | No |
| 48 | ZNF148 |  |  | 2756 | 0.049 | 0.0654 | No |
| 49 | DLD |  |  | 2811 | 0.047 | 0.0613 | No |
| 50 | ANKRD26 |  |  | 2918 | 0.043 | 0.0511 | No |
| 51 | SLX4IP |  |  | 2930 | 0.043 | 0.0519 | No |
| 52 | MED13 |  |  | 3145 | 0.037 | 0.0288 | No |
| 53 | BIRC2 |  |  | 3207 | 0.035 | 0.0234 | No |
| 54 | MFN1 |  |  | 3376 | 0.031 | 0.0054 | No |
| 55 | NUP43 |  |  | 3389 | 0.031 | 0.0055 | No |
| 56 | LCOR |  |  | 3397 | 0.031 | 0.0061 | No |
| 57 | MAGI2 |  |  | 3399 | 0.031 | 0.0075 | No |
| 58 | NDUFS1 |  |  | 3461 | 0.029 | 0.0018 | No |
| 59 | FBXO21 |  |  | 3474 | 0.029 | 0.0018 | No |
| 60 | SF3B2 |  |  | 3509 | 0.028 | -0.0009 | No |
| 61 | SLC29A4 |  |  | 3579 | 0.026 | -0.0076 | No |
| 62 | DCUN1D1 |  |  | 3632 | 0.025 | -0.0125 | No |
| 63 | UBR1 |  |  | 3634 | 0.025 | -0.0114 | No |
| 64 | CPEB4 |  |  | 3702 | 0.023 | -0.0181 | No |
| 65 | ZBTB44 |  |  | 3728 | 0.022 | -0.0200 | No |
| 66 | PLS3 |  |  | 4021 | 0.015 | -0.0531 | No |
| 67 | DCLK2 |  |  | 4033 | 0.014 | -0.0537 | No |
| 68 | GLCCI1 |  |  | 4180 | 0.010 | -0.0702 | No |
| 69 | SEPT2 |  |  | 4315 | 0.006 | -0.0854 | No |
| 70 | WAC |  |  | 4366 | 0.005 | -0.0910 | No |
| 71 | SLK |  |  | 4414 | 0.004 | -0.0963 | No |
| 72 | TNRC6A |  |  | 4595 | -0.001 | -0.1171 | No |
| 73 | FRS2 |  |  | 4756 | -0.005 | -0.1354 | No |
| 74 | PLXNA4 |  |  | 4834 | -0.007 | -0.1440 | No |
| 75 | EPHB2 |  |  | 5132 | -0.014 | -0.1778 | No |
| 76 | ASAP1 |  |  | 5241 | -0.017 | -0.1895 | No |
| 77 | KIFAP3 |  |  | 5280 | -0.018 | -0.1931 | No |
| 78 | CFDP1 |  |  | 5304 | -0.018 | -0.1949 | No |
| 79 | SP3 |  |  | 5332 | -0.019 | -0.1971 | No |
| 80 | GMFB |  |  | 5412 | -0.021 | -0.2052 | No |
| 81 | PPFIA1 |  |  | 5489 | -0.024 | -0.2129 | No |
| 82 | FRG1 |  |  | 5594 | -0.027 | -0.2237 | No |
| 83 | WDR48 |  |  | 5655 | -0.029 | -0.2293 | No |
| 84 | NEK1 |  |  | 5800 | -0.033 | -0.2444 | No |
| 85 | TIAM1 |  |  | 5861 | -0.035 | -0.2497 | No |
| 86 | FBXW7 |  |  | 5922 | -0.036 | -0.2550 | No |
| 87 | RCOR3 |  |  | 6277 | -0.049 | -0.2937 | No |
| 88 | CORO1C |  |  | 6385 | -0.052 | -0.3036 | No |
| 89 | DIMT1 |  |  | 6410 | -0.053 | -0.3039 | No |
| 90 | PBX1 |  |  | 6413 | -0.053 | -0.3016 | No |
| 91 | FAM76A |  |  | 6435 | -0.054 | -0.3015 | No |
| 92 | RAB8A |  |  | 6682 | -0.065 | -0.3269 | No |
| 93 | BBX |  |  | 6717 | -0.067 | -0.3277 | No |
| 94 | COPS8 |  |  | 7011 | -0.082 | -0.3578 | No |
| 95 | RND3 |  |  | 7179 | -0.092 | -0.3728 | No |
| 96 | YWHAG |  |  | 7373 | -0.106 | -0.3901 | No |
| 97 | OAZ2 |  |  | 7374 | -0.106 | -0.3850 | No |
| 98 | INTS10 |  |  | 7415 | -0.110 | -0.3845 | No |
| 99 | SLC25A3 |  |  | 7543 | -0.122 | -0.3934 | No |
| 100 | RAB13 |  |  | 7553 | -0.122 | -0.3887 | No |
| 101 | NFE2L2 |  |  | 7651 | -0.133 | -0.3936 | No |
| 102 | PCNP |  |  | 7665 | -0.135 | -0.3887 | No |
| 103 | POMP |  |  | 7786 | -0.150 | -0.3955 | Yes |
| 104 | CWC15 |  |  | 7821 | -0.155 | -0.3921 | Yes |
| 105 | CNP |  |  | 7852 | -0.158 | -0.3881 | Yes |
| 106 | FEZ1 |  |  | 7903 | -0.166 | -0.3860 | Yes |
| 107 | TOX3 |  |  | 7951 | -0.177 | -0.3830 | Yes |
| 108 | PSMD7 |  |  | 7976 | -0.181 | -0.3772 | Yes |
| 109 | DENR |  |  | 8031 | -0.189 | -0.3745 | Yes |
| 110 | SOX4 |  |  | 8123 | -0.209 | -0.3751 | Yes |
| 111 | JUND |  |  | 8150 | -0.214 | -0.3679 | Yes |
| 112 | ACTG1 |  |  | 8389 | -0.289 | -0.3817 | Yes |
| 113 | RPL10 |  |  | 8426 | -0.304 | -0.3715 | Yes |
| 114 | HMGN1 |  |  | 8432 | -0.308 | -0.3574 | Yes |
| 115 | ACTB |  |  | 8444 | -0.312 | -0.3439 | Yes |
| 116 | PFN2 |  |  | 8503 | -0.342 | -0.3344 | Yes |
| 117 | RPS11 |  |  | 8516 | -0.345 | -0.3194 | Yes |
| 118 | PTMA |  |  | 8523 | -0.348 | -0.3035 | Yes |
| 119 | CAMTA1 |  |  | 8540 | -0.357 | -0.2884 | Yes |
| 120 | SRP9 |  |  | 8553 | -0.365 | -0.2725 | Yes |
| 121 | RPS3 |  |  | 8598 | -0.400 | -0.2586 | Yes |
| 122 | MYL6B |  |  | 8618 | -0.422 | -0.2407 | Yes |
| 123 | RPS27 |  |  | 8638 | -0.441 | -0.2220 | Yes |
| 124 | RPL23A |  |  | 8649 | -0.454 | -0.2015 | Yes |
| 125 | RPS23 |  |  | 8690 | -0.506 | -0.1821 | Yes |
| 126 | CFL1 |  |  | 8698 | -0.516 | -0.1584 | Yes |
| 127 | PFN1 |  |  | 8732 | -0.624 | -0.1326 | Yes |
| 128 | SH3BGRL3 |  |  | 8746 | -0.785 | -0.0968 | Yes |
| 129 | TMSB4X |  |  | 8748 | -0.920 | -0.0532 | Yes |
| 130 | FTL |  |  | 8751 | -1.124 | -0.0000 | Yes |
Table: GSEA details [plain text format]

  

Fig 2: RICHARDS\_NOWAKOWSKI\_SCIENCE\_2017\_NIN4\_UPREG: Random ES distribution      
 Gene set null distribution of ES for **RICHARDS\_NOWAKOWSKI\_SCIENCE\_2017\_NIN4\_UPREG**
